# Parametric Physically Grounded Rendering of Otoscopic Morphology for Synthetic Medical Image Generation

**DOI:** 10.3390/jimaging12090424

**Published:** 2026-09-09

**Authors:** William Keustermans, Djibriel Barrie, Sam Van der Jeught

**Affiliations:** InViLab, Electromechanics Department, Faculty of Applied Engineering, University of Antwerp, 2020 Antwerpen, Belgiumsam.vanderjeught@uantwerpen.be (S.V.d.J.)

**Keywords:** physically grounded rendering, depth estimation, shape reconstruction, synthetic data generation, shape modeling

## Abstract

The tympanic membrane (TM) is a thin, semi-transparent structure whose morphology and optical appearance provide important diagnostic cues. In the early stages of middle-ear pathology, subtle shape and compliance alterations may precede overt clinical signs, making them valuable early indicators of disease. Such structural changes are difficult to assess reliably using conventional (micro-)otoscopy, which lacks quantitative depth information and is operator-dependent. Data-driven monocular image analysis could enable quantitative assessment of TM geometry and compliance, but the limited availability of annotated three-dimensional datasets constrains the development of these methods. At the same time, realistic simulations of TM appearance remain challenging due to its complex reflectance and transmission behavior. The present study focuses on physiologically healthy tympanic membranes, which provide the baseline anatomical and optical model required before subtle pathological changes can be investigated. This work introduces a parametric physically grounded rendering model of the structures visible during otoscopy: the tympanic membrane, ear canal, and malleus–incus complex. Implemented in the open-source software Blender™ using procedural geometry nodes and physically motivated shaders, the framework generates anatomically plausible three-dimensional geometries via statistical parameter sampling and controlled mesh deformation. Optical appearance is simulated using a computationally efficient layered shading model based on literature-derived tissue reflectance, transmission, and scattering properties. A camera–projector setup models both conventional white-light otoscopy and structured-light imaging, enabling the generation of paired intensity images and corresponding depth maps. The proposed framework establishes a physically grounded representation of the human ear and enables a controllable, extensible modeling pipeline for virtual training, biomechanical finite element analysis, and synthetic data generation for supervised learning.

## 1. Introduction

The tympanic membrane (TM) is a thin, semi-transparent structure that separates the external ear canal (EC) from the middle ear (ME) cavity. Its morphology and optical appearance contain valuable diagnostic information. According to the World Health Organization, almost sixty percent of hearing impairment in children could be prevented if common causes were addressed at the primary healthcare level [1]. Otoscopy is the visual examination of the EC and TM and is used to diagnose a wide range of common ME-related diseases. It is a common procedure and a key skill for all clinicians, including general practitioners and pediatricians [2]. Conventional otoscopy provides only a 2D view of the TM, limiting the ability to quantify subtle shape changes [3]. However, clinically relevant pathologies such as Eustachian tube (ET) dysfunction and otitis media are correlated with TM deformation and retraction [4], motivating the development of 3D otoscopic imaging techniques [5,6,7]. In addition, dynamic measurements of TM shape provide a non-invasive marker of ET function as patients with obstructive ET dysfunction are unable to adequately equalize ME pressure, leading to insufficient aeration, TM retraction, and potential damage to the ossicular chain. Chronic impairment of ME pressure equalization has a reported prevalence of approximately 4.6% among adults and 6.1% among children in the United States [8,9]. Quantifying TM surface deformation during maneuvers (e.g., Valsalva) may therefore offer an objective means of assessing ME pressure regulation and ET function.

Accordingly, the present work first focuses on physiologically healthy TMs. Establishing a reliable baseline model of healthy anatomy and optical appearance is a necessary first step for future investigation of early-stage disease, where morphological and optical deviations may be subtle.

Developing supervised methods for quantitative otoscopy requires more than diagnostic class labels: each structured-light image must be paired with an accurate, spatially registered 3D surface of the same TM in the same physiological state. Acquiring such ground-truth data in vivo is particularly difficult. The TM’s position restricts the working distance and camera–projector baseline, may cause partial occlusion, and leaves no room for an independent reference instrument [5,6,7]. Physiological motion and small changes in probe pose can disrupt the pixel-level correspondence between sequential measurements. Ex vivo techniques can provide detailed surface measurements [10,11,12], but do not capture the TM in the same physiological state or under the same clinical imaging conditions. This problem is especially pronounced for early-stage ME pathology, where geometric changes may be subtle and routine clinical annotations generally consist of diagnostic categories or qualitative observations rather than dense, metric depth maps. Consequently, no real-world otoscopic datasets currently provide pixel-aligned pairs of structured-light fringe images and ground-truth depth maps. This limitation directly motivates the present framework, which generates controlled image, fringe, and depth data from the same parametric 3D anatomy with exact pixel-level correspondence.

Structured light profilometry (SLP) is an established technique for ex vivo TM surface reconstruction [10,11,12]. It uses projected light patterns to reconstruct the 3D shape of the surface. The basic setup consists of a projector and a camera positioned at a relative angle to each other. When the projector illuminates the object with a known pattern (e.g., sinusoidal fringes), the camera records the geometrically deformed pattern. From these measured deformations, the surface depth can be reconstructed (e.g., through triangulation). However, classical multi-shot approaches require multiple sequential projected patterns and are therefore sensitive to synchronization constraints and physiological motion artifacts inherent to in vivo TM imaging.

In contrast, single-shot deep learning (DL) SLP aims to reconstruct a depth map from a single image of a deformed fringe pattern, enabling higher imaging speed. With deep learning now widely adopted in medical image analysis for learning hierarchical image representations directly from data [13], this paradigm can be extended to directly estimate surface geometry from fringe images. Van der Jeught & Dirckx [14] first demonstrated that an end-to-end network mapping a deformed fringe map to a height field can achieve high reconstruction accuracy, while implicitly learning intermediate processing steps such as noise filtering, geometric calibration, and phase unwrapping. In their work, synthetic height maps were generated using a random surface generator combined with an analytical forward model, in which sampled height fields were mapped to their corresponding sinusoidal fringe images via a height-dependent phase shift, enabling highly efficient generation of large training datasets. Within this simplified framework, fringe patterns exhibit uniform contrast and are perturbed only by additive homogeneous noise.

Real otoscopic fringe images, however, are shaped by a combination of non-idealities, including tissue-dependent reflectance and specular highlights on the tympanic membrane, leading to spatially varying fringe visibility and intensity components not explained by geometry alone. This domain gap compromises 3D reconstruction performance in real-world clinical settings.

Physics-based rendering (PBR) offers a way to simulate optical image formation using anatomically consistent morphologies, material models, and controlled illumination. By simulating light transport, PBR enables the generation of realistic images of biological tissues under diverse illumination conditions, including structured light projection. Visually realistic synthetic medical images can also be generated using purely data-driven models, notably generative adversarial networks (GANs) and diffusion models, which primarily learn image distributions rather than explicitly modeling image-formation physics [15,16]. Recent comparative work on scientific image synthesis by Sordo et al. [17] reported high perceptual quality and structural coherence for StyleGAN and high realism for diffusion-based models, while also emphasizing that visual fidelity does not necessarily guarantee scientific accuracy. Within otoscopy, Suresh and colleagues [18] demonstrated that StyleGAN2-ADA can produce realistic otoscopic images when trained solely on clinical data, and Camalan et al. [19] more recently reported improved multi-class otoscopy classification performance when StyleGAN3-generated images were used for data augmentation. These approaches are therefore valuable for reproducing visual appearance and expanding limited datasets. However, they do not inherently provide metric 3D ground truth or guarantee consistency with a specified camera, illumination, or structured-light configuration. In contrast, the present parametric PBR framework explicitly controls anatomy, material properties, and imaging conditions and generates physically consistent paired fringe images and depth maps from the same underlying 3D surface. This paired geometric ground truth is crucial for downstream supervised machine learning (ML) applications. In addition, unlike GAN- or diffusion-based synthesis, the proposed parametric framework does not require a training image dataset and allows targeted generation of specific anatomical configurations, including underrepresented cases. We present a procedural physically grounded model of the medial part of the EC and TM implemented in the open-source software Blender™ (version 4.5 LTS, Blender Foundation, Amsterdam, The Netherlands), which combines morphometric sampling, procedural mesh generation, physically motivated shading, and structured light rendering within a single framework. For clarity, it should be noted that the term virtual otoscopy refers to computed tomography (CT)-based endoluminal rendering of middle-ear anatomy, and should not be confused with the present work, which focuses on physically grounded simulation of optical image formation during digital otoscopy. To the authors’ knowledge, this work is the first to integrate anatomical variability, procedural mesh generation, and physically grounded rendering into a single unified computational framework for generating otoscopic images with controllable anatomical and optical variability. The contributions of this work are:

1. A parametric modeling framework combining procedural mesh generation with statistically informed anatomical variability, enabling synthesis of realistic middle ear geometries.
2. An integrated PBR setup that couples procedural material shaders with configurable camera and projector models, supporting both white-light otoscopy simulation and structured light projection.
3. A controlled synthetic data generation methodology that produces physically consistent paired outputs (fringe images and depth maps), facilitating downstream supervised ML applications.

The present study focuses on the development and initial image-level evaluation of this simulation framework. Downstream reconstruction performance and sim-to-real transfer are beyond the scope of the present work.

## 2. Materials and Methods

The proposed methodology consists of four main components. First, a sampling module generates morphological parameters using statistical distributions derived from literature data. These parameters are then used in a procedural geometry module to generate the EC, TM, and ossicle meshes. Next, a procedural shading module assigns material properties to all structures. Finally, a rendering module simulates the camera–projector configuration for structured-light imaging and captures the corresponding depth maps.

### 2.1. Anatomical and Optical Characterization

The realism of the generated synthetic dataset strongly depends on whether the sampled anatomical and optical parameters remain within physiological ranges. The proposed pipeline, therefore, relied on anatomical and biomechanical literature and reports to construct statistical distributions. Together, these sources provide a quantitative basis for generating anatomical variability consistent with natural inter-subject variation.

The ear canal is typically oval in cross-section and follows an S-shaped course; it is commonly divided into three segments [20]. An otoscope primarily visualizes the most medial section, from the second bend onwards, where the EC is lined with thin skin, and where the outermost layer, the epidermis, continues seamlessly onto the TM. The absence of ceruminous glands and hair follicles in this region allowed these structures to be excluded from the model [21]. Given that the morphology of the EC varies with age, sex, ethnicity, and laterality, anthropometric measurements derived from tomographic data were used to parameterize the geometric variability of the canal [22].

**Figure 1:**
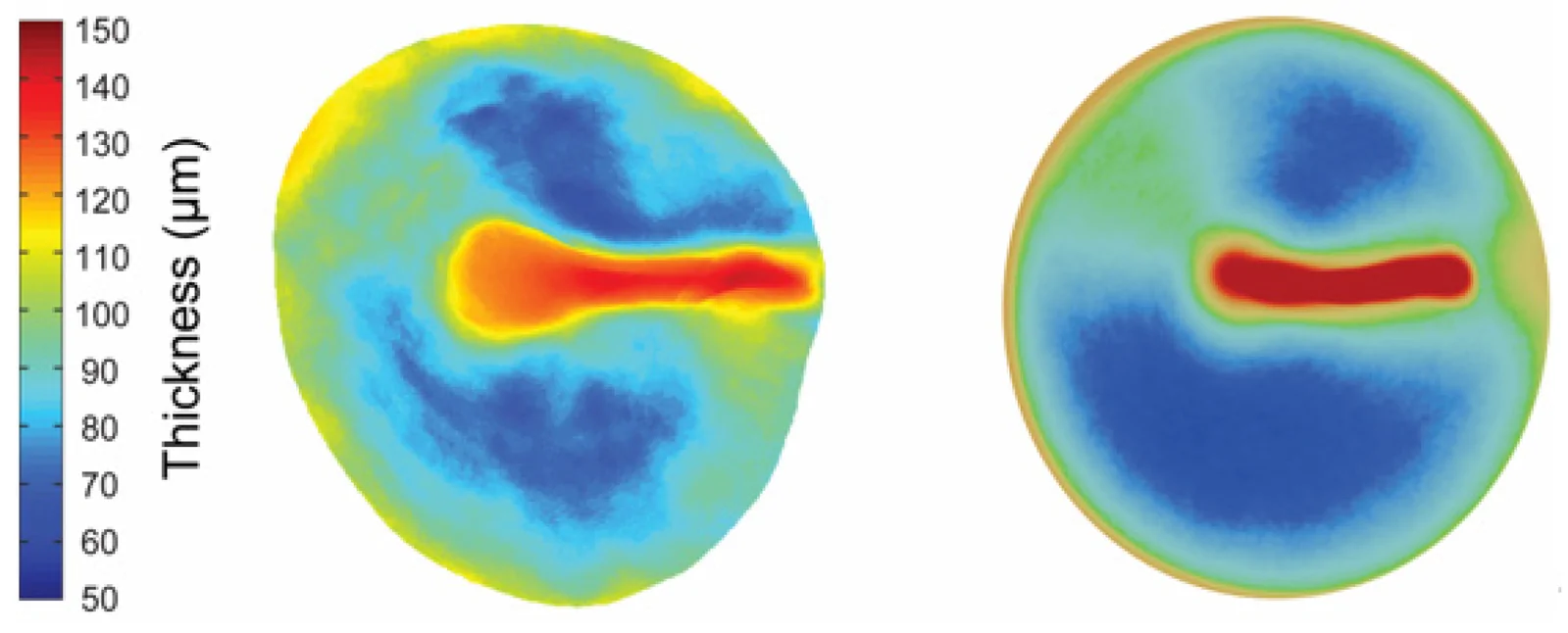
The shortest-distance thickness map [27] (**Left**) gives an orientation-independent estimate of local thickness and served as a reference template for constructing a continuous and adaptive thickness distribution map for our model ((**Right**), presented on a simple oval shape). To facilitate visual comparison, the umbo position in our model was shifted off-center, and an IMC instance with a larger malleus handle region was selected to better align with the reference template.

The main parameters defining the conical-shaped TM include the horizontal and vertical diameters and umbo position. Umbo depths of approximately 1.4 to 2 mm are reported by Volandri et al. [23]. In Lenarz et al. [24], the horizontal and vertical TM diameters were extracted from high-resolution clinical imaging data of 50 patients. A multivariate normal distribution was fitted to these data to capture their joint variability, yielding mean values of approximately 9.7 mm (horizontal) and 8.8 mm (vertical). The TM has a non-uniform thickness, ranging from 30 µm to 150 µm, with an inter-individual variability in the mean thickness of approximately 74 µm [23,25]. The annular ring is a thickened circumferential rim that surrounds nearly the entire perimeter of the tympanic membrane. In cross-section, the ring exhibits a wedge-shaped profile. Its width is not uniform but varies along the circumference, ranging on average from approximately 748 µm inferiorly to 113 µm posteriorly [26]. To incorporate realistic spatial thickness variations, a full-field thickness map was implemented based on optical coherence tomography (OCT) measurements [27]. In that study, thickness distributions from five healthy human tympanic membranes were reconstructed, registered, and combined into an averaged thickness map. The shortest-distance thickness map (Figure 1, left) was selected to obtain an orientation-independent estimate of local thickness and served as a reference template for constructing a continuous thickness distribution. This distribution was implemented as an adaptive thickness map that is automatically adjusted to each procedurally generated TM instance (Figure 1, right), ensuring consistent anatomical features such as local thickening along the malleus handle.

The eardrum is tilted with respect to the EC, with its lateral surface facing anteriorly and inferiorly. This orientation, together with the shape of the eardrum, results in a visible cone of light in the anteroinferior quadrant during otoscopic examination. Ranges of TM angular orientation were obtained from the literature [23,28,29].

At its medial surface, the tympanic membrane is attached to the handle of the malleus, which, starting from the umbo, extends upwards and anteriorly when viewed through the external ear canal. A gamma distribution was fitted to the literature-reported quantiles by Todd [28] using quantile matching and combined with rejection sampling to generate realistic values for the angle between the malleus handle and the vertical axis. Because the malleus and incus are often partially visible through the translucent TM, anatomical variability in ossicular geometry was incorporated. A statistical shape model of the first two auditory ossicles, discussed in Section 2.3, was therefore integrated into the framework.

Beyond geometry, realistic image formation requires accurate modeling of light–tissue interactions.

**Figure 2:**
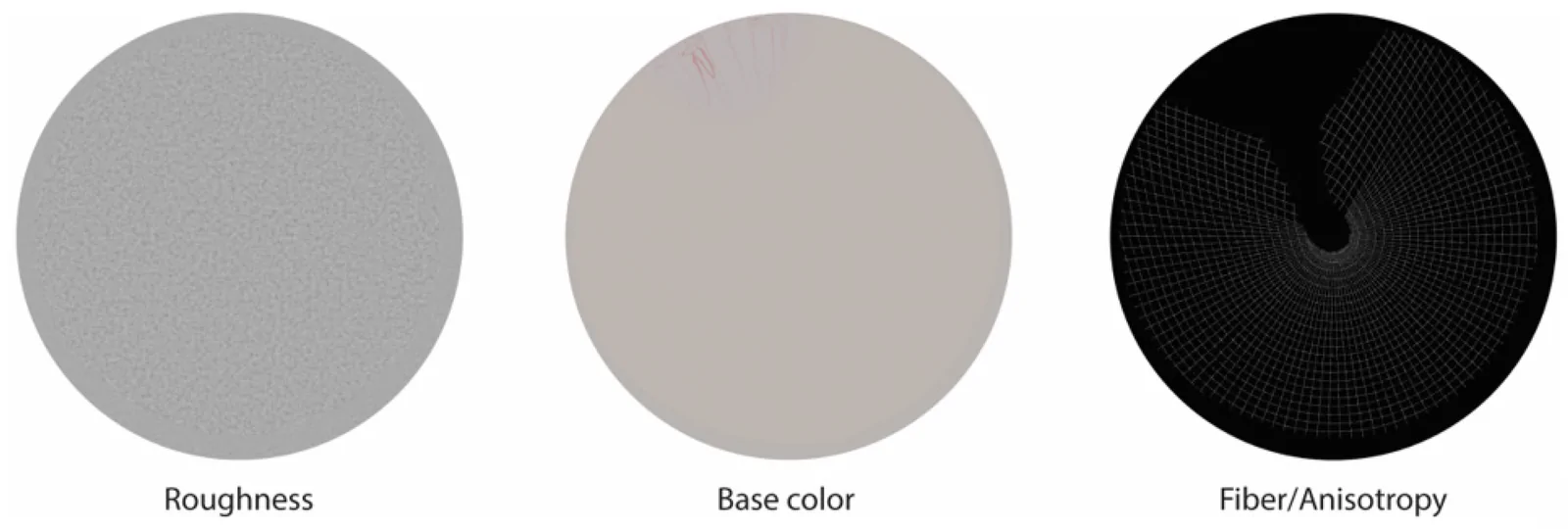
(Fine-scale variations become visible upon magnification) Adaptive texture maps used in the TM shading model. (**Left**) A specular roughness map introduces regional variations in surface reflectance, with an overall lower roughness on the annular ring. (**Middle**) The base color map captures the characteristic grayish-to-reddish appearance (note the subtle difference with the annular ring). (**Right**) An anisotropy map is used to modulate the scattering behavior across the pars tensa.

The tympanic membrane is semi-translucent and typically exhibits a grayish or reddish hue depending on the region (Figure 2), with its visual appearance arising from a combination of specular reflection, diffuse scattering, and subsurface light transport. To model these effects in a physically grounded manner, optical properties such as refractive index, scattering coefficient, anisotropy factor, and absorption coefficients were derived from the literature.

Refractive index measurements of the TM in the visible range have not been reported in the literature; therefore, a representative value of 1.44 measured at 1050 nm was adopted [27]. Although this value was obtained outside the visible spectrum, it is consistent with reported visible-spectrum refractive indices of constituent soft tissues. Wave-length-dependent measurements of epidermal tissue yield a mean refractive index of approximately 1.44 across the visible spectrum, while representative values for dermis and mucosa are approximately 1.38 and 1.37, respectively [30,31]. The eardrum is divided into two main regions: the pars tensa and the pars flaccida. The former constitutes the largest region and consists of a three-layer structure composed (from lateral to medial) of the epidermis, the lamina propria, and the mucosa. Anisotropy is defined as the mean cosine of the scattering angle and was assumed to be approximately constant in the visible spectrum. Based on reported tissue optical properties, an anisotropy value of approximately 0.78 was adopted for the tympanic membrane [30]. Moreover, the adopted value of 0.78 lies within the relatively narrow range of approximately 0.75–0.80 reported or estimated for the constituent tissue types in the visible spectrum [30]. In addition, the lamina propria of the pars tensa contains organized radial and circumferential collagen fibers, which not only influence the mechanical behavior of the membrane but also affect light scattering. Therefore, an anisotropy (fiber) map was introduced to modulate scattering behavior across the TM surface, allowing regions with pronounced fiber structures to exhibit directionally different light interaction compared to fiber-poor areas such as the pars flaccida (Figure 2). Optical absorbance is determined by the relative volume fractions of key chromophores, including blood, water, and melanin. Goblet and colleagues measured the TM’s optical absorbance in four human samples [32]. Scattering parameters were derived from reported optical properties of biological tissue layers, i.e., epidermis, dermis, and mucosa, using values summarized by Jacques [33]. The use of dermal scattering properties as a proxy for the lamina propria is supported by previous optical work in which TM scattering was likewise considered comparable to dermis. Measured two-way optical loss through the TM yielded a best-fit scattering coefficient of approximately 155/cm [34]. The equivalent scattering properties were subsequently derived from the optical properties and relative thicknesses of the individual TM layers, rather than adopting a single generic tissue value. No additional correction term for Fresnel reflection at the internal interfaces between the constituent TM tissue layers was included when deriving the effective scattering properties, as these contributions were considered negligible relative to the overall scattering.

### 2.2. Statistical Shape Model of the First Two Hearing Ossicles

To synthesize anatomically plausible renderings of the TM together with the middle-ear structures visible through its translucent surface, a representation of the malleus–incus (incudomallear) complex (IMC) was incorporated behind each sampled TM instance. The ossicles model is derived from a previously developed statistical shape model (SSM) constructed from clinical cone-beam CT (CBCT) scans (150 µm voxel size) of 100 patients [35]. Both temporal bones were acquired where available. After exclusion of diseased ears, 147 healthy middle ears remained, of which 123 could be successfully brought into dense correspondence and were included in the final SSM. Further details on data selection, segmentation, and SSM construction are provided in Soons et al. [35]. The ossicles were segmented and smoothed and brought into dense correspondence via elasticity-modulated surface registration [36].

In the present work, the ossicles were pose-normalized using a similarity-based Procrustes alignment before principal component analysis (PCA). As a result, the derived PCA space captures shape variability while excluding global translation, rotation, and scale. For each generated sample, the ossicle instance is positioned and scaled relative to the sampled umbo location and depth, and size of the TM, to ensure anatomically consistent alignment with the TM geometry.

**Figure 3:**
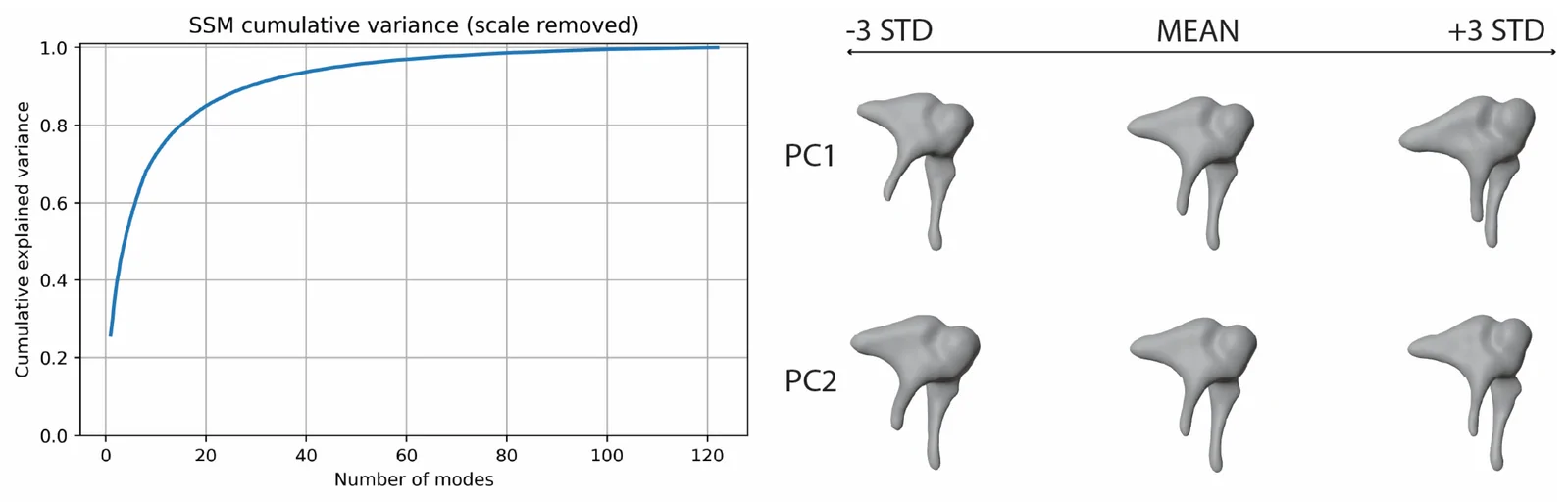
(**Left**) Compactness of the IMC shape model. (**Right**) Visualization of the mean shape and the first two orthogonal modes (principal components) that capture the anatomical variation in the dataset. Realistic variability of the ossicular geometry is achieved by sampling an IMC instance from the statistical shape space for each rendering.

The IMC SSM provides a mean shape and orthogonal modes describing the principal anatomical variation in the dataset. Lower-order modes represent global size-like deformations, while higher orders capture localized shape changes. A limited number of modes suffices to explain most of the variance; principal components (PCs) 1–27 account for approximately 90 percent of the total variation (Figure 3).

### 2.3. Procedural Geometry

In the following, the procedural pipeline is presented. A total of 15 parameters (not counting the SSM’s PCs) and a random seed were implemented to control variability. The set of morphological attributes defining the global geometry includes the TM diameters, umbo depth and position, relative TM orientation, and high- and low-frequency TM surface variations. These parameters are summarized in Table 1.

The pipeline starts from a cylindrical template geometry with unit radius and height. This choice ensures a watertight and consistent mesh topology in which the tympanic membrane and ear canal are generated as a single continuous surface. Starting from a closed primitive reduces the need for Boolean operations and mesh stitching, which are prone to numerical instabilities and self-intersections in procedural generation workflows, improving robustness across large-scale randomized sampling.

**Figure 4:**
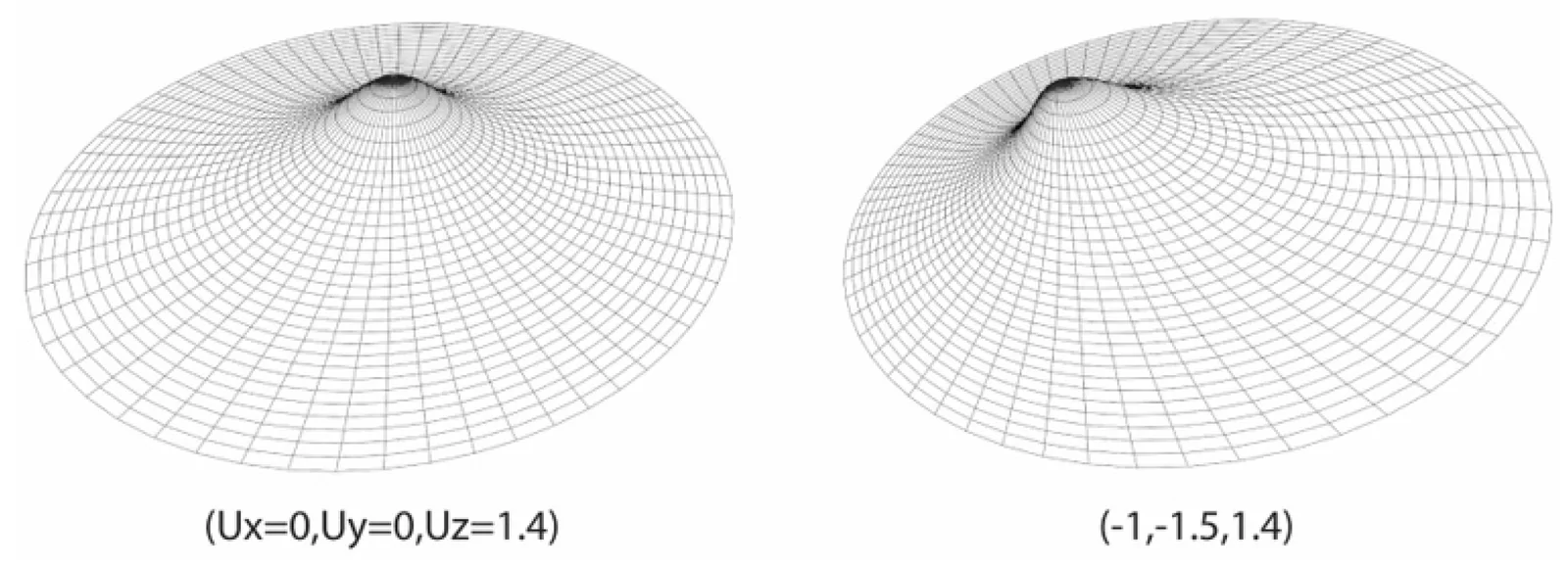
Two instances of the cylinder’s upper surface conically deformed. Shifting the umbo position introduces asymmetry (**Right**). Notice how the umbo consistently corresponds to the central vertex.

The base shape is then scaled to match the sampled elliptical TM boundary defined by the horizontal and vertical diameters and extruded to the ear canal length. The upper surface of the cylinder is subsequently transformed into an initial TM-like shape by applying a conical deformation and introducing asymmetry by shifting the umbo position (Figure 4). The mesh is retopologized by computing ray–ellipse intersections to maintain the umbo as the central vertex.

**Figure 5:**
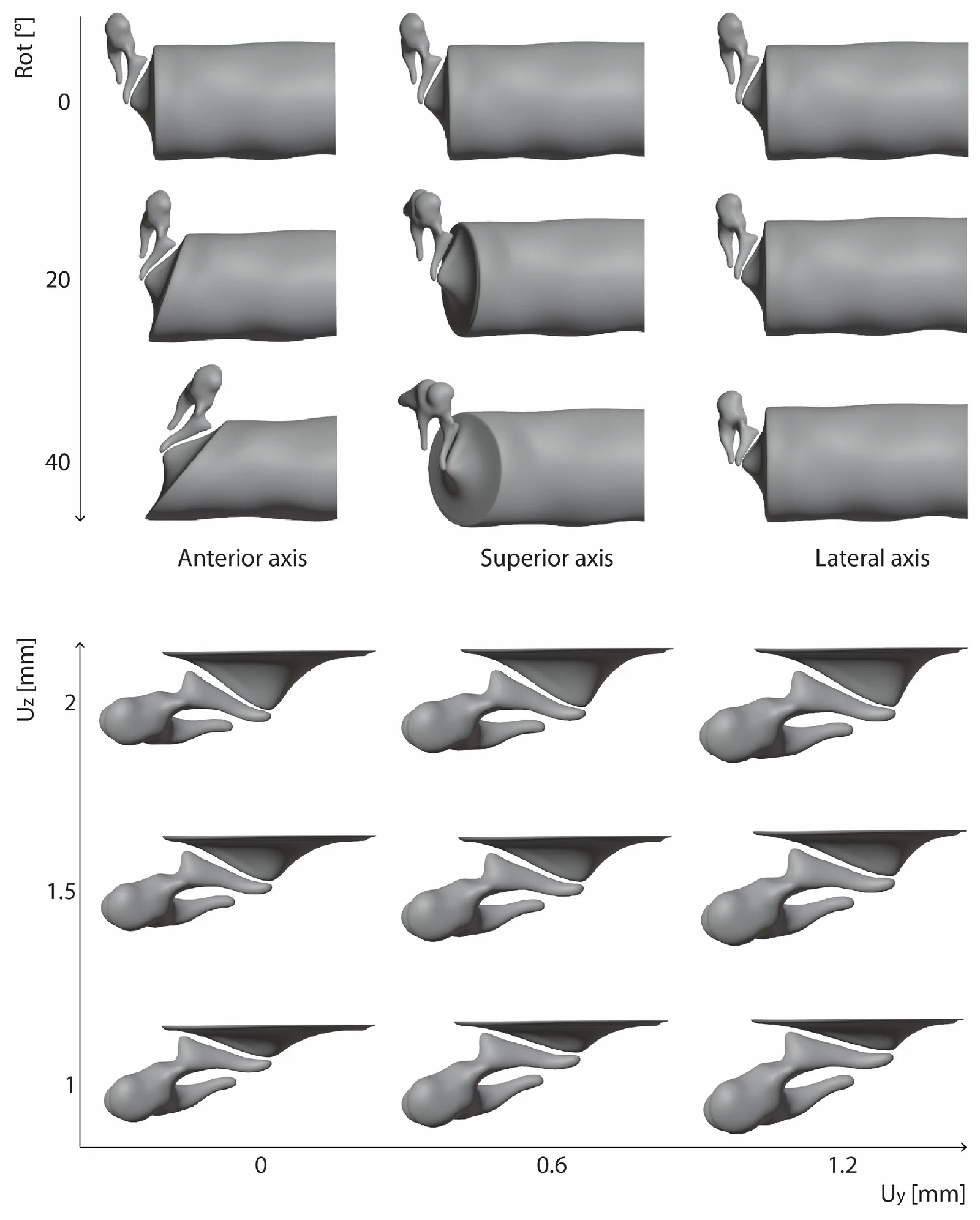
(**Top**) Rotations of the TM around the anterior, superior, and lateral axes. (**Bottom**) Paired parameter variation of the umbo’s y- and z-coordinates. The IMC (here represented by the mean geometry) undergoes rigid co-variation and scales proportionally with the TM geometry.

To account for the medial attachment of the malleus handle, a manubrium region is defined with reduced deformation along this axis. Next, the membrane is rotated to represent the TM’s tilt relative to the ear canal (Figure 5, top). The annular ring is modeled by shaping a circumferential rim of non-uniform width around the membrane perimeter. Low- and high-frequency Perlin noise components (controlled by a random seed) are applied to the TM surface to mimic biological irregularities and natural variability. The medial part of the ear canal, i.e., the portion between the TM and the second canal bend, is generated procedurally, including controlled variations in curvature and cross-sectional shape. Finally, an instance of the first two ossicles is sampled, scaled, and positioned behind the tympanic membrane (Figure 5, bottom).

**Figure 6:**
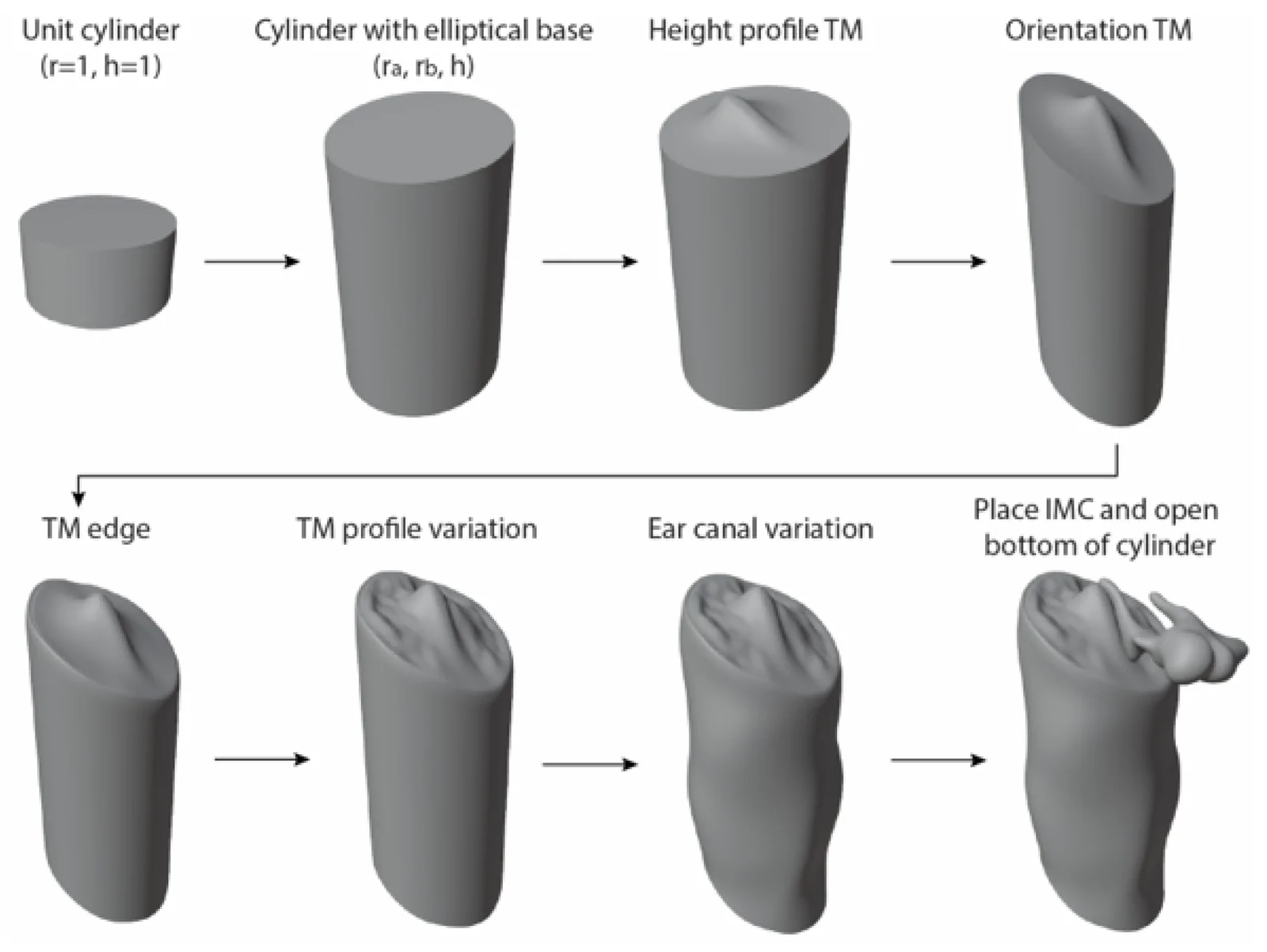
Procedural mesh generation: pipeline that transforms the set of sampled morphological parameters into the corresponding shape in approximately 180 ms.

The entire procedural pipeline requires approximately 180 ms per sample and enables the generation of a large number of unique, anatomically plausible surfaces without manual modeling (Figure 6). By combining parameter sampling and random seed control, the model supports a wide range of variability in TM and EC geometry, facilitating the generation of large-scale synthetic datasets.

### 2.4. Procedural Shading

The tympanic membrane is a multilayer biological structure composed of the epidermis, lamina propria, and mucosa. The base-color component was defined to reproduce the characteristic grayish-to-reddish appearance of healthy TMs and was combined with a procedural vascular component. Rather than introducing vascularization only as a global color contribution, the model reproduces the characteristic spatial organization of TM vasculature, with vessels predominantly extending radially from the peripheral region and along the manubrial region. This vascular contribution is spatially constrained, thereby preserving regional differences such as the pars flaccida. Procedural texture patterns, including Voronoi-based structures, are used to introduce local variation in vessel distribution and appearance.

Its observed appearance results from a combination of specular surface reflection, internal scattering and absorption, diffuse reflection and transmission, and back-scattered contributions from the ME cavity. Together, these mechanisms produce the characteristic otoscopic appearance, including glossy highlights, a reddish-grey coloration, and partial visibility of ME structures. To realistically reproduce these effects, a physically motivated shader was developed that captures both surface interaction (specular reflection) and internal light transport (diffuse reflection and transmission). Considering the balance between physical accuracy and computational efficiency, a single-point shading approximation was adopted. While this approach does not explicitly simulate full volumetric light transport, literature-based optical parameters provide visually faithful results at practical computation times. Accordingly, although Cycles provides physically based light transport, the tissue representation used here is physically grounded rather than a rigorous radiative-transfer model.

**Figure 7:**
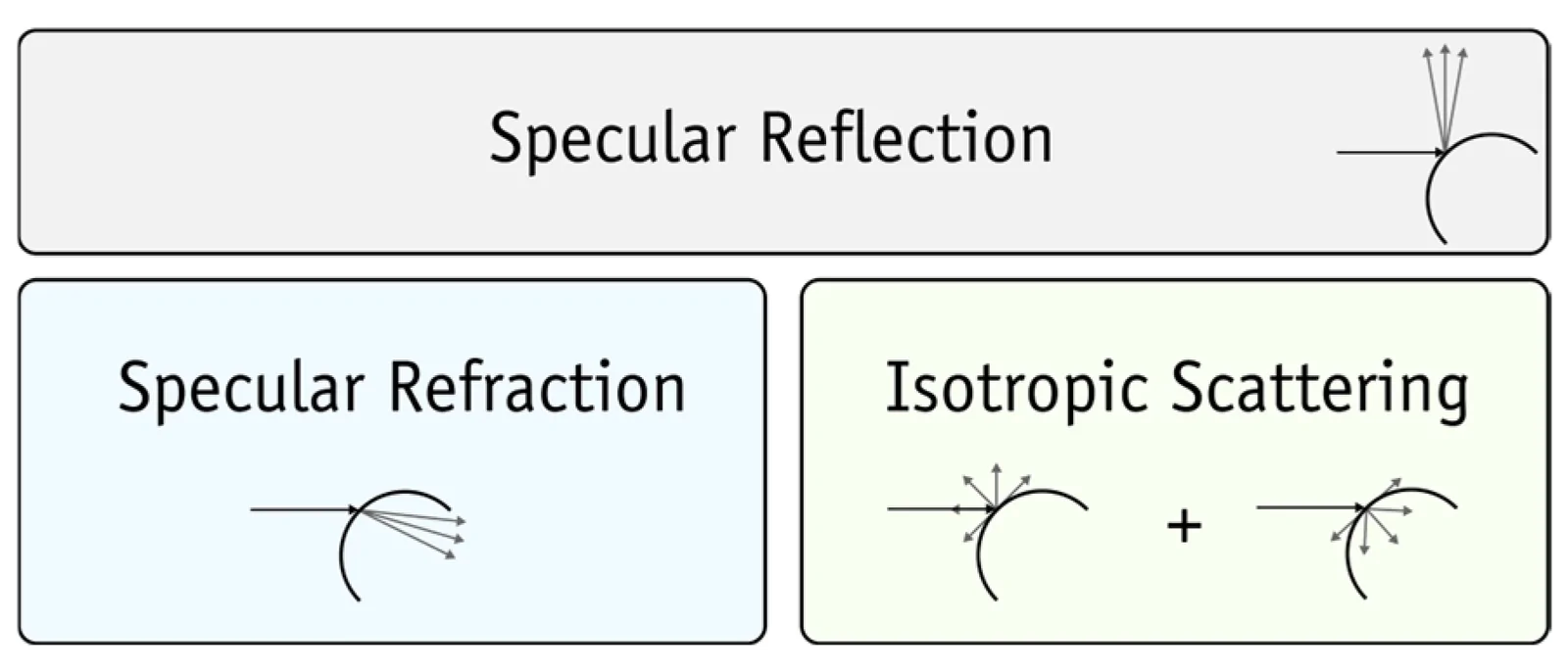
The TM’s single-point shader architecture. The vertical direction represents layered material components, while the horizontal direction represents mixing between terms.

The shader follows a layered mixed-shader formulation. A reflective top coat is used to approximate surface reflection and microfacet behavior, while a transmissive substrate models internal scattering and absorption within the membrane. In contrast to the neglected internal inter-layer Fresnel terms described in Section 2.1, the Fresnel interaction at the external air–TM surface is explicitly represented in the top layer of the shader. The first layer determines the relative amount of reflection and transmission using the Fresnel equation based on the TM’s refractive index. The second layer approximates subsurface light transport by mixing two simplified components: an isotropic scattering component and a transmitted component. This structure enables independent control of surface roughness and transmission roughness, providing a good approximation of subsurface scattering while remaining computationally efficient. Figure 7 illustrates the shader architecture, where the vertical direction represents layered material components and the horizontal direction represents mixing between shader terms.

To obtain a computationally efficient weighting between forward transmission and diffuse scattering, a transport-inspired approximation was used. The coefficient μ in this expression denotes the scattering coefficient $\mu_{s}$ and does not include absorption. The latter is handled separately in the material model. The product $\left(1-g\right)\cdot \mu$ corresponds to the reduced scattering coefficient $\mu_{s}^{’}$. For a membrane thickness t, an effective forward-transmission weight T was defined $T=I/I_{0}=e^{-\left(1-g\right)\cdot µ\cdot t}$, where g is the anisotropy factor representing the mean cosine of the scattering angle. The expression provides a computationally efficient mapping from transport optical thickness to the relative shader contributions. In the implemented shader, the transmitted component was weighted by T and the isotropic scattering component by (1 − T).

To capture fine spatial heterogeneity, additional procedural texture nodes (e.g., Voronoi patterns) were used to introduce subtle local variation in scattering and absorption properties. Furthermore, a bump map was included to create small-scale surface irregularities that generate highlight distortions consistent with clinical otoscopic observations.

The ear canal was modeled using a simplified surface shader. Since the tympanic membrane is the primary imaging target, the canal material was approximated as moderately reflective soft tissue with lower roughness, enabling the generation of characteristic specular highlights and realistic secondary reflections on the tympanic membrane.

### 2.5. Camera and Fringe Projector

Rendering is performed using Blender’s Cycles ray-tracing engine. Render parameters, such as resolution and sample count, were optimized to favor visual realism while maintaining practical computation times. To extract the corresponding depth map, Blender’s Z-pass was enabled, which outputs a distance-to-surface image in the camera’s reference frame.

Structured-light acquisition was simulated using a physically based fringe projector model implemented in Blender, based on an open-source projector project [37]. This projector model mimics a real optical projection system by incorporating intrinsic projection parameters such as throw ratio, projector resolution, and lens shift. A sinusoidal fringe texture consisting of vertically oriented intensity-modulated stripes was projected onto the scene to emulate fringe projection profilometry. The projection source was configured with an optical output power representative of the intended otoscopic acquisition conditions, rather than using artificially high-intensity fringe illumination. By rendering the deformed fringe pattern on the tympanic membrane surface together with its corresponding depth map, paired training samples were generated with exact pixel-wise correspondence between intensity and geometry.

Right-ear instances were generated by mirroring the anatomical geometry before rendering rather than by image mirroring, thereby preserving physically consistent illumination and viewing conditions. White-light otoscopic images contain multiple laterality cues arising not only from anatomical features, such as malleus orientation (pointing toward the face) and umbo position, but also from optical cues, including illumination direction and specular reflections. Although image mirroring may appear valid at first glance (since prominent features such as the cone of light visually shift to anatomically plausible positions), it implicitly reverses instrument-dependent imaging characteristics (the effective handedness of the imaging device). While these inconsistencies may already be noticeable in white-light otoscopy (where illumination and imaging are largely co-axial), they become critical in structured-light simulations, where surface geometry is encoded through projector–camera triangulation. While switching from a left to a right ear corresponds to a mirrored geometry, the projector–camera configuration remains unchanged. Image mirroring would invert the deformation of the projected fringes, making them inconsistent with the fixed projector–camera setup. In other words, this would produce fringe maps that cannot occur when using the real acquisition device [6].

### 2.6. Quantitative Image Fidelity

Image fidelity was assessed using an otoscopy-specific Natural Image Quality Evaluator (NIQE) model [38] and the Kernel Inception Distance (KID) [39]. The real reference dataset comprised 105 images of healthy tympanic membranes obtained from two publicly available sources: 91 images from the healthy/normal class of the Otoscopic Image Dataset (Kaggle) [40] and 14 images labeled as normal tympanic membranes in the Otolaryngology Image Atlas [41]. The Otoscopic Image Dataset is released under a CC0 1.0 Public Domain dedication. The Otolaryngology Image Atlas is publicly accessible online. The Kaggle subset was manually curated to remove duplicate and mirrored images, images with substantial cerumen, and severely out-of-focus acquisitions, resulting in 91 retained images. A pool of 1000 white-light synthetic otoscopic renderings was generated for comparison.

To reduce sensitivity to dataset-specific framing, all real and synthetic images underwent identical preprocessing. Images were center-cropped to a square with a side length equal to 60 percent of the shorter image dimension to reduce peripheral borders and field-of-view differences, and resized to 288 × 288 pixels using Lanczos resampling. No intensity or color normalization was applied, as this would suppress appearance differences that form part of the fidelity assessment.

For NIQE, the real images were split into training/test sets comprising 80 and 25 images while preserving the source distribution, respectively (Kaggle: 69/22; Otolaryngology Image Atlas: 11/3). To prevent information leakage, remaining strongly similar frames identified using a 256-bit difference hash (Hamming distance ≤ 10) were assigned to the same split. An otoscopy-specific NIQE model was fitted to the 80 real training images using the fitniqe function in MATLAB R2024a (MathWorks, Natick, MA, USA), with non-overlapping 96 × 96-pixel blocks and a sharpness threshold of 0. The fitted model was subsequently evaluated on the 25 held-out real images and on 105 synthetic images randomly selected from the synthetic pool using a fixed seed. NIQE scores quantify deviation from the feature statistics learned from real healthy otoscopic images, with lower values indicating closer correspondence to the real-image reference distribution.

For KID, all 105 real images were compared with the same fixed-seed subset of 105 synthetic images. Images were processed using the pretrained Inception-v3 network [42] implemented in TorchMetrics 1.9.0 [43] and torch-fidelity 0.4.0 [44], with internal resizing to 299 × 299 pixels, and 2048-dimensional feature representations were extracted. KID was calculated as the unbiased squared maximum mean discrepancy using a third-degree polynomial kernel. The mean and standard deviation were estimated over 100 reproducible resamples containing 50 images from each distribution. As an internal reference for the variability expected within the real dataset, a real–real baseline was calculated using the same procedure, with two disjoint random subsets of 50 real images in each resample. Lower KID values indicate greater similarity between the compared feature distributions.

## 3. Results

This section presents representative white-light and structured-light renderings generated with the proposed framework, followed by a quantitative comparison of synthetic and real otoscopic images.

The procedural geometry generation is computationally efficient, producing a complete tympanic membrane, ear canal, and ossicular geometry in approximately 180 ms per sample. Rendering subsequently dominates the total computation time and depends strongly on parameters such as image resolution and sample count. At a resolution of 1024 × 1024 pixels with 1000 samples, rendering required approximately 80 s per image. Renderings at 512 × 512 pixels were generated in under 20 s, whereas 256 × 256 images required only a few seconds, depending on GPU performance (NVIDIA^®^ GeForce™ GTX 1080, Santa Clara, CA, USA) [6].

### 3.1. Digital Otoscope Simulation: White-Light Rendering of Healthy TMs

**Figure 8:**
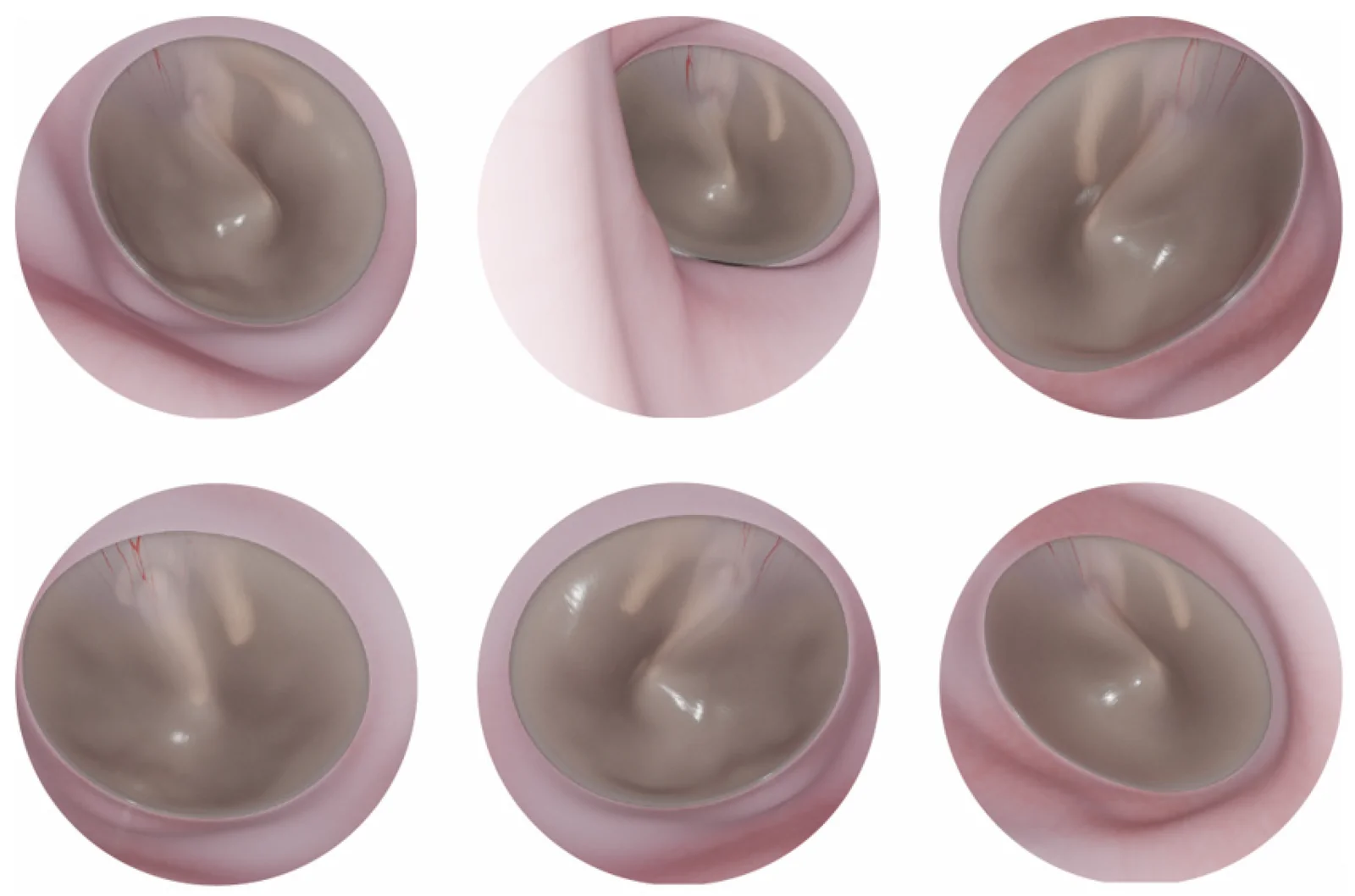
Examples of white-light otoscopic renderings generated with the framework described in Section 2. Specular reflections, including the characteristic cone of light, can be observed, as well as partial occlusion of the tympanic membrane by the ear canal (as can be the case in real-life otoscopic examination). Note also the ossicular morphology variations behind the TM, driven by the PCs of the statistical shape model.

**Figure 9:**
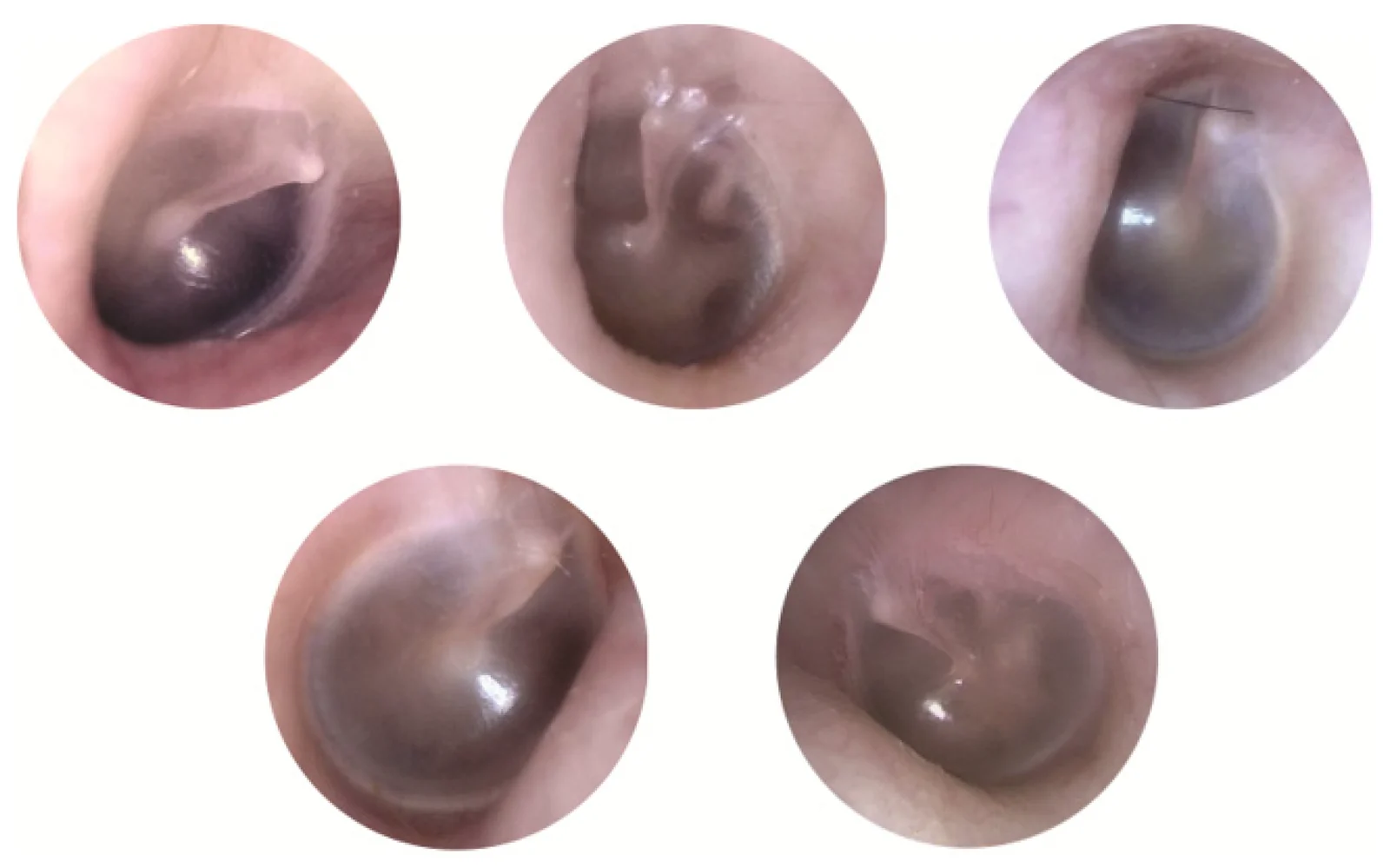
Edited otoscopic images of the Otolaryngology Image Atlas labeled as normal by otolaryngologists [41].

Representative white-light renderings are shown in Figure 8. The framework generated otoscopic renderings under uniform illumination that reproduce characteristic visual cues, including specular highlights (e.g., the cone of light in the anteroinferior quadrant and arc-shaped highlights tracing the annular rim) and partial transparency of the TM with visibility of middle-ear structures. Some synthesized external auditory canals present realistic occlusion and field-of-view constraints, reproducing the typical tunnel-like otoscopic appearance (Figure 9). Variations in ossicular morphology, driven by the principal components of the statistical shape model, were perceptible through the TM.

### 3.2. Three-Dimensional Otoscope Simulation: Fringe Projection Rendering

**Figure 10:**
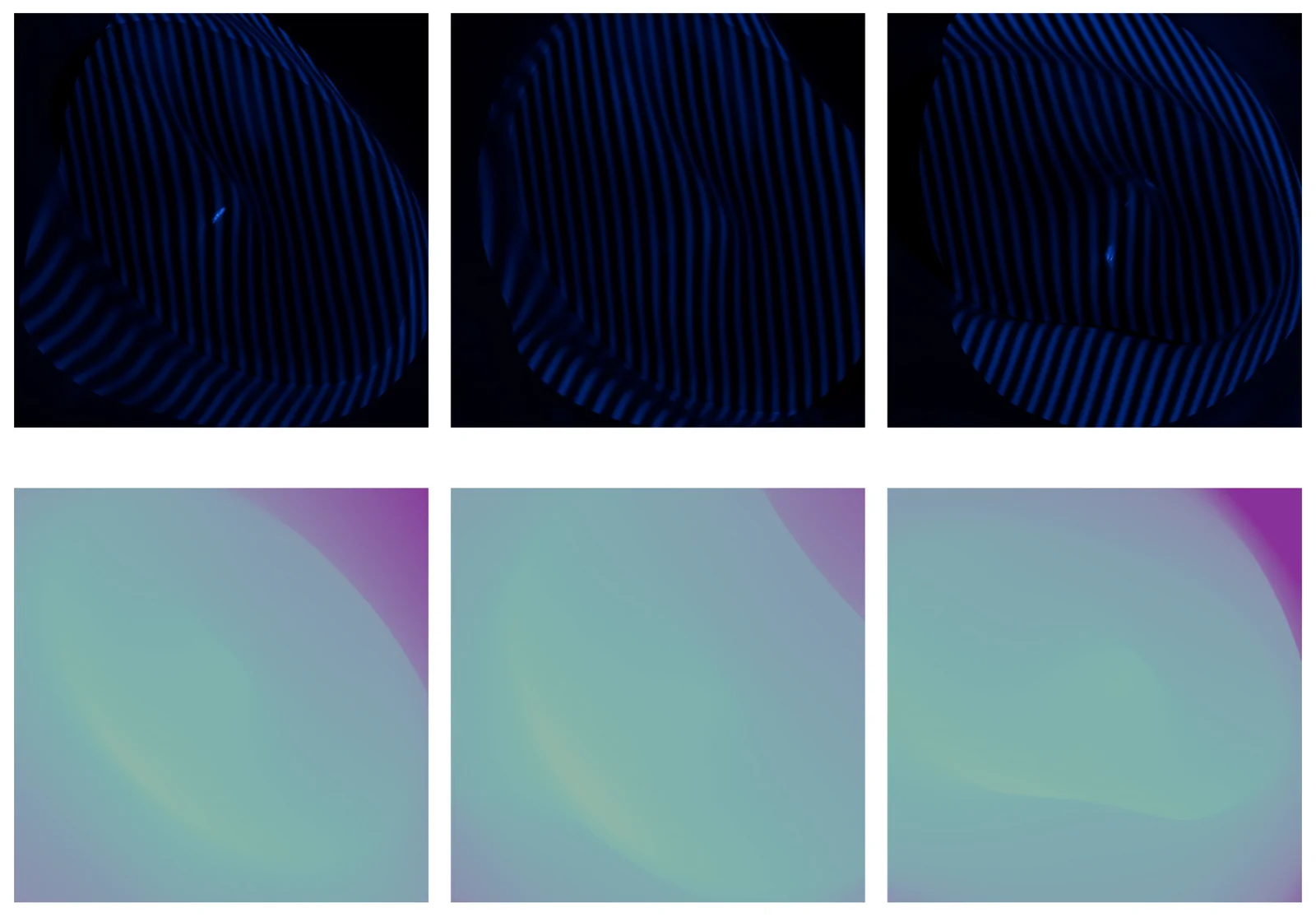
(**Top**) Blue-light fringe projection renderings generated with the framework described in Section 2. No white navigation light is used to maximize fringe contrast. Specular highlights occur together with dim diffuse fringe reflections. In some renderings, partial occlusion of the projected light field by the ear canal results in non-illuminated regions on the TM. The TM shows lower overall return intensity than the ear canal wall, with additional local lower contrast due to light reflected from the ossicles. (**Bottom**) Corresponding depth maps. Depth is color-coded using the Viridis colormap, with purple hues representing smaller depth values (closer to the camera) and progressively greener hues representing larger depth values (farther from the camera).

Using the camera–projector setup, the system renders deformed fringe patterns on the tympanic membrane surface while simultaneously extracting the corresponding depth map using Blender’s Z-Pass. This yields paired fringe images and ground-truth depth fields with exact pixel-wise correspondence (Figure 10). The rendered fringe patterns reflect the underlying TM geometry and exhibit acquisition-related effects such as partial occlusion by the ear canal, non-illuminated regions, and local intensity and contrast variations caused by differences in tissue reflectance and light contributions from the ossicles. The selected projector output also resulted in relatively low overall image intensity and reduced fringe visibility on the TM.

### 3.3. Quantitative Image Fidelity

The held-out real images yielded an otoscopy-specific NIQE score of 2.71 ± 0.96 (mean ± SD, *n* = 25), whereas the synthetic images yielded a substantially higher score of 11.16 ± 1.69 (*n* = 105). This indicates that the low-level image statistics of the current synthetic renderings differ noticeably from those learned from real healthy otoscopic images. The real–synthetic KID was 0.2652 ± 0.0100, compared with 0.00022 ± 0.00223 for the real–real baseline across matched resamples. The feature-space distance between real and synthetic images therefore exceeded the variability observed between disjoint subsets of the real-image dataset by a large margin. Together, NIQE and KID identify a clear appearance-domain gap between the current white-light renderings and real healthy otoscopic images. Neither metric assesses anatomical correctness or the utility of the geometrically aligned fringe–depth pairs, which require separate validation.

## 4. Discussion

### 4.1. Model Performance

The present results focus on physiologically healthy tympanic membranes. This choice is motivated by the clinical observation that advanced middle-ear pathology is often readily identifiable using conventional otoscopy once structural changes become pronounced. The greater diagnostic challenge lies in early-stage or preventive assessment, where only subtle morphological changes, such as early retraction pockets or localized dynamic changes in TM compliance, may be present while visual appearance remains largely normal. A realistic optical model of healthy anatomy is therefore essential for studying early detection strategies. Nonetheless, the proposed framework could be extended to simulate pathological conditions through parameter adjustments. For example, inflammatory states such as otitis media could be reproduced by modifying base color distributions toward increased global redness, enhancing vascular prominence, and increasing optical opacity. In cases of otitis media with effusion, outward bulging of the TM due to middle-ear overpressure could be simulated in Blender through deformation functions implemented via so-called float curves. These deformation profiles could be parameterized along and perpendicular to the manubrium to allow region-specific shape variation. Furthermore, by incorporating a spatial constraint map for the manubrial region, the model would reproduce the reduced displacement along the handle of the malleus during deformation, consistent with reported biomechanical observations [45].

The qualitative real-versus-synthetic comparison (Figure 8 and Figure 9) and the quantitative image-fidelity analyses reported in Section 3.3 provide complementary assessments of similarity between synthetic and real otoscopic images. The quantitative comparison further shows that, despite reproducing several characteristic otoscopic features qualitatively, a substantial image-domain gap remains between the current synthetic renderings and real healthy otoscopic images. Both the otoscopy-specific NIQE score and KID clearly separated the synthetic and real distributions, with the real–synthetic KID exceeding the real–real baseline by a large margin. This indicates that visual plausibility at the level of characteristic anatomical and optical features does not yet translate into full statistical similarity to real acquisitions.

Several factors may contribute to this discrepancy. The present model deliberately approximates aspects of the complete imaging chain, including tissue light transport, spectral behavior, camera optics, and sensor characteristics. In addition, the real reference images originate from different acquisition systems and therefore contain device-dependent variations in illumination, color response, sharpness, compression, and noise that are not explicitly reproduced by the current renderer. The reported metrics should therefore be interpreted as measures of the present appearance-domain gap rather than as direct measures of anatomical or physical accuracy. Importantly, reducing this gap is expected to be relevant for future sim-to-real transfer and provides a quantitative target for subsequent refinement of the rendering pipeline.

A more direct physical validation of the image-formation model would require geometry-matched real and synthetic acquisitions. Specifically, the same TM and EC geometry would need to be measured independently and subsequently rendered under corresponding imaging conditions. Such validation remains challenging as the appearance of an otoscopic image depends on the underlying three-dimensional geometry of the TM and EC. Such validation would require topographic or tomographic measurement combined with optical imaging of the same ear under corresponding acquisition conditions, allowing a synthetic rendering to be generated from the measured geometry and directly compared with its real counterpart. Clinical (cone-beam) CT does not resolve the TM sufficiently due to its sub-millimeter thickness, while OCT systems capable of capturing detailed TM geometry remain costly and are not widely available. Future availability of patient-specific three-dimensional measurements or reconstructions, including those obtained with the DL-based 3D otoscope described in Section 4.2, could facilitate geometry-matched validation of the proposed rendering framework. Structured perceptual assessment by otologists would provide an additional complementary validation of visual realism and remains an important direction for future work.

Importantly, the framework provides exact pixel-wise correspondence between the rendered structured-light images and their underlying depth maps, which is a prerequisite for supervised reconstruction training. Whether models trained on these synthetic data achieve sufficient reconstruction accuracy and generalize to real otoscopic acquisitions remains to be established experimentally.

### 4.2. Applications

Although otoscopy can be practiced on peers or patients with minimal discomfort, effective performance requires precise coordination between instrument handling and visual interpretation, which makes supervision and structured feedback during training challenging [2]. Moreover, studies have shown that general practitioners and medical students often demonstrate only moderate diagnostic proficiency in otoscopy [2,46], underscoring the need for improved training strategies. Simulation-based approaches have therefore been introduced, ranging from web-based 3D visualization platforms [47] to more advanced systems capable of tracking otoscope motion and providing automated feedback [48]. Evidence suggests that such simulation-based training can enhance learner confidence and improve diagnostic accuracy [49,50].

Within this context, a PBR model could serve as a digital training platform for ear–nose–throat physicians. When combined with otoscopic hardware (e.g., a handheld shell equipped with an inertial measurement unit), the system could support interactive training scenarios in which physicians practice navigating the ear canal and interpreting otoscopic views. Unlike static image databases, a procedural simulator can generate a large amount of anatomical variation, including challenging cases such as unusual TM orientations, partial occlusions, or narrow canal geometries. However, to enable such interactive training scenarios, the model first has to be coupled to a real-time rendering engine. Rasterization-based engines such as Blender’s EEVEE are better suited, as they can provide sufficiently realistic visual feedback at interactive frame rates, in contrast to offline ray-tracing engines such as Cycles.

The combined EC, TM, and IMC geometry can also serve as an input mesh for finite element modeling (FEM) of the middle-ear transfer function and tympanic membrane deformation under pressure variations. By sampling different anatomical shapes, FEM studies could investigate population-level variability in mechanical response. However, for biomechanical simulations, the tympano-mallear connection (also called the manubrial fold) has to be included. Notably, its geometry and dimensions exhibit substantial inter-individual variability and may significantly influence ME mechanics, as reported by [51].

**Figure 11:**
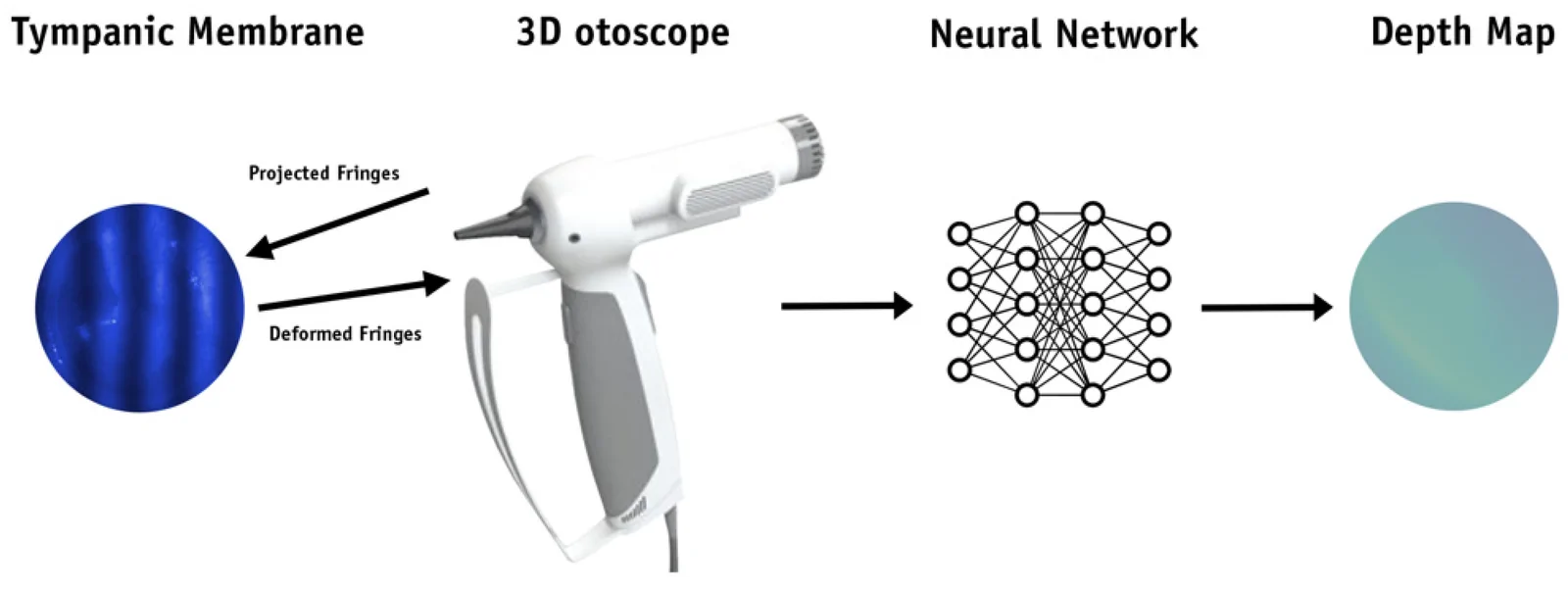
Schematic overview of the single-shot DL-based 3D otoscope’s working principle. A fringe pattern is projected onto the TM, and the resulting deformed fringes are captured by the imaging sensor. This image is processed by a deep neural network, which generates the corresponding depth map.

The primary motivation of this work, however, was synthetic dataset generation for supervised learning of single-shot SLP (Figure 11). DL-based SLP requires a large dataset containing paired fringe images and ground-truth depth maps. Such a dataset is very difficult and costly to acquire in vivo. A physically grounded simulation provides a controllable and scalable alternative, supporting rapid iteration in both optical design and DL development. The present work should therefore be regarded as a proof-of-concept framework for generating the paired synthetic data required for this downstream application. The actual reconstruction performance of networks trained on these data, including their generalization to real otoscopic acquisitions, remains to be established experimentally.

### 4.3. Current Limitations

The current camera model is an ideal pinhole camera with effectively unlimited depth of field. In contrast, real otoscopes exhibit depth-of-field limitations imposed by their compact lens and aperture configuration. This discrepancy can be reduced by optimizing the hardware design for increased depth of field, as discussed in Keustermans et al. [6]. Alternatively, future work could incorporate lens-based camera models to more accurately reproduce optical defocus and aberrations, further improving image realism. Such a lens-based model can be implemented without volumetric shaders by representing the individual refractive interfaces explicitly. For spherical lens elements, each optical surface (e.g., two surfaces for a singlet and three unique surfaces for a cemented doublet) can be constructed from its radius of curvature and clear aperture using the spherical sag equation. Aspherical surfaces can analogously be defined using the standard conic/aspheric sag equation and their corresponding conic and higher-order aspheric coefficients. Successive surfaces have to be positioned according to the specified center thicknesses. Each refractive interface can then be assigned a transmissive glass shader using the relative refractive index of the two adjacent media. Correct surface-normal orientation and relative refractive index are essential to reproduce the intended direction and magnitude of refraction in Blender. The resulting irradiance distribution can be recorded on a Lambertian sensor plane and viewed using an orthographic camera. This lens-based configuration allows rays originating from the same object point to pass through different positions of the finite lens aperture and subsequently converge onto the image plane. It can therefore reproduce finite depth of field and geometric optical aberrations. Such physically based lens simulations may, however, substantially increase rendering time. In particular, focusing light from an emissive object through refractive surfaces onto a sensor constitutes a challenging caustic light-transport problem for Blender’s unidirectional path-tracing engine, Cycles. More advanced sampling or light-transport strategies may therefore be required to make this approach computationally practical. Renderers supporting bidirectional path tracing or light tracing may be better suited for this extension.

Blender Cycles is also not a spectral renderer and approximates wavelength-dependent behavior using RGB color channels. Biological tissue optics, however, can exhibit strong wavelength dependence, particularly due to hemoglobin absorption and subsurface scattering. As a result, the simulated color response may deviate from true physical behavior under certain illumination conditions. The perceived appearance of light interacting with tissue depends not only on the material properties but also on the characteristics of the observer, whether this is the human visual system, a camera sensor, or another detector. In principle, physically based rendering should therefore be performed spectrally, i.e., by simulating light transport across the full wavelength range and converting the resulting spectrum to RGB values only at the final stage. However, for practical rendering purposes, RGB-based rendering is commonly used. Although this approximation is not strictly physically accurate, it still produces realistic results when the underlying optical parameters are chosen carefully. When optical properties such as refractive index, absorption, and scattering coefficients are reported as wavelength-dependent functions, they must ideally be converted to RGB values by accounting for the spectral sensitivity of the sensor. This conversion can be achieved using a technique called color matching [52]. In the present model, however, these wavelength-dependent parameters were approximated at three representative wavelengths corresponding to the RGB channels: 650 nm (red), 550 nm (green), and 450 nm (blue).

Both the idealized camera model and the RGB approximation may contribute to the sim-to-real gap in downstream learning applications. The absence of finite-depth-of-field effects may result in differences in spatial sharpness, local contrast, and fringe visibility compared with real otoscopic acquisitions, whereas the RGB approximation may introduce discrepancies in wavelength-dependent tissue color and contrast. Networks trained exclusively on synthetic data could therefore learn image characteristics that are not fully representative of the real acquisition system.

A related limitation concerns uncertainty in the optical input parameters for which direct human TM measurements in the visible spectrum are unavailable. The refractive index, anisotropy, and scattering parameters used here should therefore be interpreted as physically motivated approximations rather than definitive visible-spectrum optical properties of the human TM. Within the present shader, uncertainty in refractive index primarily affects the Fresnel balance between surface reflection and transmission, whereas uncertainty in scattering and anisotropy affects the balance between forward transmission and diffuse scattering and consequently TM translucency, image contrast, and the visibility of underlying middle-ear structures.

Another limitation concerns the heterogeneous sample sizes underlying the anatomical priors. The full-field TM thickness template was derived from only five healthy tympanic membranes, whereas the diameter distribution was based on measurements from 50 patients. Consequently, these priors may not fully capture population-level inter-individual variability, particularly with respect to spatial thickness patterns.

Another limitation of the proposed approach is that, although the geometry generation is based on morphometrical data, it does not constitute a full-blown SSM and therefore does not explicitly constrain the global shape to the manifold of natural variation. For deep learning applications, this is not necessarily a limitation, as the primary requirement is that the training dataset spans sufficient variability to ensure robust generalization. Nevertheless, an SSM-based representation of the human tympanic membrane would be a valuable extension, particularly in the context of biomechanical FEM analysis. Such a model could better capture correlated anatomical variations and may provide insight into whether biological variability is constrained in specific structural regions that are critical for normal hearing function.

A further acquisition-related limitation is that the present simulations do not explicitly model sensor noise or motion blur. Since both effects depend strongly on the specific imaging hardware, exposure settings, and acquisition conditions, future work will parameterize these degradations from measurements of the physical otoscope rather than applying arbitrary noise or blur levels. Their relevance can subsequently be assessed through the robustness and reconstruction accuracy of networks trained on the resulting synthetic data.

## 5. Conclusions

This work introduced a parametric rendering framework of the structures visible during otoscopy, integrating controllable geometry, physically grounded appearance modeling, and a configurable virtual camera–projector setup within a unified workflow. The model reproduces characteristic otoscopic visual cues and enables the generation of geometrically consistent image–depth pairs for both white-light and structured-light imaging. By combining statistical shape variation with physically grounded shading and acquisition modeling, the framework addresses the scarcity of annotated three-dimensional otoscopic data while maintaining interpretability and control over the underlying geometry and image formation process. Although further refinement of lens modeling and spectral realism is warranted, the proposed approach establishes a flexible computational basis for synthetic data generation, quantitative otoscopy research, and future integration with data-driven reconstruction methods. The present study establishes the proof-of-concept simulation framework and its initial image-level evaluation; the downstream performance of reconstruction models trained on the generated data and their sim-to-real transfer remain to be quantitatively validated.

## Figures and Tables

**Table 1:** Summary of the 15 procedural geometry parameters used to control TM and EC variability, including their sampling distributions, implemented ranges, and literature sources. The minor and major TM diameters are sampled jointly from a multivariate normal distribution with covariance 0.4794 mm^2^. Parameters indicated as model-specific were assigned bounded procedural ranges because no direct quantitative population distributions were available. The PC weights of the SSM and random seeds are not included in the table. (^1^) Diameter perpendicular to the manubrium. (^2^) Diameter along the manubrium. (^3^) Local TM prominence along the manubrium, corresponding to the malleolar stria. (^4^) Rotation signs follow a right-handed coordinate convention. Because left- and right-ear geometries are mirrored, the sign corresponding to the same anatomical orientation is ear-side-dependent; the indicated positive/negative ranges therefore preserve the anatomical orientation of the manubrium toward the face and the anterior tilt of the TM. (^5^) Gamma distribution with rejection sampling over the anatomically plausible range.

Parameter	Distribution	Implemented Range/Parameters	Literature Source
Minor TM diameter ^1^ [mm]	Bivariate normal (joint with major TM diameter)	μ = 9.6781 σ = 0.7233	Lenarz et al. [24]
Major TM diameter ^2^ [mm]	Bivariate normal (joint with minor TM diameter)	μ = 8.8435 σ = 0.9974	Lenarz et al. [24]
Umbo depth [mm]	Normal	μ = 1.60 σ = 0.11	Volandri et al. [23]
X-position apex with respect to TM center [mm]	Uniform	−1.5 to 1.5	Model-specific, no population distribution available
Y-position apex with respect to TM center [mm]	Uniform	−1.5 to 1.5	Model-specific, no population distribution available
Manubrial strength ^3^	Uniform	0 to 1	Model-specific procedural range
Manubrial width ^3^	Uniform	0 to 1	Model-specific procedural range
TM low-frequency surface variation: amplitude	Uniform	0 to 1	Model-specific procedural setting
TM low-frequency surface variation: scale	Uniform	0 to 0.3	Model-specific procedural range
TM high-frequency surface variation: amplitude	Uniform	0 to 1	Model-specific procedural range
TM high-frequency surface variation: scale	Uniform	0.3 to 1	Model-specific procedural range
Ear-canal surface variation: amplitude	Uniform	0 to 1	Model-specific range, EC variability motivated by Nasiri et al. [22]
TM rotation around lateral axis ^4^ [°]	Truncated transformed gamma ^5^	(–)90–(–)X, where X ~ Gamma (k = 70.95, θ = 1.01), 49.5 ≤ X ≤ 90	McManus et al. [29]
TM rotation around superior axis ^4^ [°]	Uniform	(–)10 to (–)40	[23,29]
TM rotation around anterior axis ^4^ [°]	Uniform	(–)30 to (–)45	Todd [28]

## Data Availability

The otoscopic images analyzed in this study are publicly available from the sources cited in Section 2.6. The Blender scene, source code, and associated project assets are not publicly available at this stage because they form part of an ongoing research project. Further inquiries can be directed to the corresponding author.

## Abbreviations

Following abbreviations are used in this manuscript:

CT	Computed Tomography
DL	Deep Learning
EC	Ear Canal
ET	Eustachian Tube
FEM	Finite Element Modeling
GAN	Generative Adversarial Network
IMC	Incudomallear Complex
ME	Middle Ear
ML	Machine Learning
OCT	Optical Coherence Tomography
PBR	Physics-Based Rendering
PCA	Principal Component Analysis
PCs	Principal Components
SLP	Structured Light Profilometry
SSM	Statistical Shape Model
TM	Tympanic Membrane

## References

[1] WHO (2024). Hearing Loss and the Role of Health Care Providers: Fact Sheet.

[2] Wiet G.J., Sørensen M.S., Andersen S.A.W. (2017). Otologic Skills Training. Otolaryngol. Clin. N. Am..

[3] Esposito S., Bianchini S., Argentiero A., Gobbi R., Vicini C., Principi N. (2021). New Approaches and Technologies to Improve Accuracy of Acute Otitis Media Diagnosis. Diagnostics.

[4] von Unge M., Decraemer W.F., Dirckx J., Bagger-Sjöbäck D. (1995). Shape and Displacement Patterns of the Gerbil Tympanic Membrane in Experimental Otitis Media with Effusion. Hear. Res..

[5] Bedard N., Shope T., Hoberman A., Haralam M.A., Shaikh N., Kovačević J., Balram N., Tošić I. (2017). Light Field Otoscope Design for 3D in Vivo Imaging of the Middle Ear. Biomed. Opt. Express.

[6] Keustermans W., ter Heerdt P., Dirckx J.J., Van der Jeught S. (2026). 3D Otoscope: Toward an Extra Diagnostic Dimension for Middle-Ear Related Issues. Hear. Res..

[7] Monroy G.L., Won J., Spillman D.R., Dsouza R., Boppart S.A. (2017). Clinical Translation of Handheld Optical Coherence Tomography: Practical Considerations and Recent Advancements. J. Biomed. Opt..

[8] Patel M.A., Mener D.J., Garcia-Esquinas E., Navas-Acien A., Agrawal Y., Lin S.Y. (2016). Tobacco Smoke Exposure and Eustachian Tube Disorders in US Children and Adolescents. PLoS ONE.

[9] Shan A., Ward B.K., Goman A.M., Betz J.F., Reed N.S., Poe D.S., Nieman C.L. (2019). Prevalence of Eustachian Tube Dysfunction in Adults in the United States. JAMA Otolaryngol. Head Neck Surg..

[10] Das A.J., Estrada J.C., Ge Z., Dolcetti S., Chen D., Raskar R., Choi B., Kollias N., Zeng H., Kang H.W., Wong B.J.F., Ilgner J.F., Nuttal A., Richter C.-P., Skala M.C., Dewhirst M.W. (2015). A Compact Structured Light Based Otoscope for Three Dimensional Imaging of the Tympanic Membrane. Photonic Therapeutics and Diagnostics XI.

[11] Liang J., Luo H., Yokell Z., Nakmali D.U., Gan R.Z., Lu H. (2016). Characterization of the Nonlinear Elastic Behavior of Chinchilla Tympanic Membrane Using Micro-Fringe Projection. Hear. Res..

[12] Van der Jeught S., Soons J.A.M., Dirckx J.J.J. (2015). Real-Time Microscopic Phase-Shifting Profilometry. Appl. Opt..

[13] Litjens G., Kooi T., Bejnordi B.E., Setio A.A.A., Ciompi F., Ghafoorian M., van der Laak J.A.W.M., van Ginneken B., Sánchez C.I. (2017). A Survey on Deep Learning in Medical Image Analysis. Med. Image Anal..

[14] Van der Jeught S., Dirckx J.J.J. (2019). Deep Neural Networks for Single Shot Structured Light Profilometry. Opt. Express.

[15] Yi X., Walia E., Babyn P. (2019). Generative Adversarial Network in Medical Imaging: A Review. Med. Image Anal..

[16] Dayarathna S., Islam K.T., Uribe S., Yang G., Hayat M., Chen Z. (2024). Deep Learning Based Synthesis of MRI, CT and PET: Review and Analysis. Med. Image Anal..

[17] Sordo Z., Chagnon E., Hu Z., Donatelli J.J., Andeer P., Nico P.S., Northen T., Ushizima D. (2025). Synthetic Scientific Image Generation with VAE, GAN, and Diffusion Model Architectures. J. Imaging.

[18] Suresh K., Cohen M.S., Hartnick C.J., Bartholomew R.A., Lee D.J., Crowson M.G. (2023). Generation of Synthetic Tympanic Membrane Images: Development, Human Validation, and Clinical Implications of Synthetic Data. PLoS Digit. Health.

[19] Camalan S., Langefeld C.D., Zinnia A., Moberly A.C., Gurcan M.N. (2026). StyleGAN-Based Synthetic Image Augmentation for Multi-Class Otoscopy Image Classification. Sci. Rep..

[20] Fan H., Yu S., Wang M., Li M., Chu J., Yan Y., Zhang S., Chen D., Harris-Adamson C. (2021). Analysis of the External Acoustic Meatus for Ergonomic Design: Part I—Measurement of the External Acoustic Meatus Using Casting, Scanning and Rapid Estimation Approaches. Ergonomics.

[21] Drake R.L., Vogl W., Mitchell A.W.M., Gray H. (2020). Gray’s Anatomy for Students.

[22] Nasiri M., Khabiri H., Halvani G.H., MoradiAmin M., Shamsi F., ZangiAbadi Z. (2025). Anthropometric Analysis of the External Ear Using CT Scan Image by Age, Gender, and Side Among the Iranian Population. Ergon. Des. Q. Hum. Factors Appl..

[23] Volandri G., Di Puccio F., Forte P., Carmignani C. (2011). Biomechanics of the Tympanic Membrane. J. Biomech..

[24] Lenarz T., Becker M., Warnecke A., Giesemann A., Prenzler N.K., Steinhardt U., Schurzig D. (2024). Middle Ear Anatomy and Implant Sizes: Correlates and the Need for Uniform Implant Dimensions. Front. Audiol. Otol..

[25] Yang Z., Moran Mojica M., Kim W., Oghalai J.S., Applegate B.E. (2025). Quantitative Measurement of Tympanic Membrane Structure and Symmetry with Optical Coherence Tomography in Normal Human Subjects. J. Biomed. Opt..

[26] Kassem F., Ophir D., Bernheim J., Berger G. (2010). Morphology of the Human Tympanic Membrane Annulus. Otolaryngol. Head Neck Surg..

[27] Van der Jeught S., Dirckx J.J.J., Aerts J.R.M., Bradu A., Podoleanu A.G., Buytaert J.A.N. (2013). Full-Field Thickness Distribution of Human Tympanic Membrane Obtained with Optical Coherence Tomography. J. Assoc. Res. Otolaryngol..

[28] Todd N.W. (2005). Orientation of the Manubrium Mallei: Inexplicably Widely Variable. Laryngoscope.

[29] McManus L.J., Dawes P.J.D., Stringer M.D. (2012). The Orientation of the Tympanic Membrane. Clin. Anat..

[30] Tuchin V. (2007). Tissue Optics: Light Scattering Methods and Instruments for Medical Diagnosis.

[31] Ding H., Lu J.Q., Wooden W.A., Kragel P.J., Hu X.-H. (2006). Refractive Indices of Human Skin Tissues at Eight Wavelengths and Estimated Dispersion Relations between 300 and 1600 nm. Phys. Med. Biol..

[32] Goblet M., Matin F., Lenarz T., Paasche G. (2021). Optical Absorbance of the Tympanic Membrane in Rat and Human Samples. PLoS ONE.

[33] Jacques S.L. (2013). Optical Properties of Biological Tissues: A Review. Phys. Med. Biol..

[34] MacDougall D., Rainsbury J., Brown J., Bance M., Adamson R. (2015). Optical Coherence Tomography System Requirements for Clinical Diagnostic Middle Ear Imaging. J. Biomed. Opt..

[35] Soons J.A.M., Danckaers F., Keustermans W., Huysmans T., Sijbers J., Casselman J.W., Dirckx J.J.J. (2016). 3D Morphometric Analysis of the Human Incudomallear Complex Using Clinical Cone-Beam CT. Hear. Res..

[36] Danckaers F., Huysmans T., Lacko D., Ledda A., Verwulgent S., Van Dongen S., Sijbers J. (2014). Correspondence Preserving Elastic Surface Registration with Shape Model Prior. Proceedings of the 2014 22nd International Conference on Pattern Recognition.

[37] Schell J., Schmitz P. Projector Add-on for Blender. https://github.com/Ocupe/Projectors.

[38] Mittal A., Soundararajan R., Bovik A.C. (2013). Making a “Completely Blind” Image Quality Analyzer. IEEE Signal Process. Lett..

[39] Bińkowski M., Sutherland D.J., Arbel M., Gretton A. Demystifying MMD GANs. Proceedings of the International Conference on Learning Representations (ICLR).

[40] UCI Machine Learning Otoscopic Image Dataset. Kaggle. https://www.kaggle.com/datasets/ucimachinelearning/otoscopic-image-dataset.

[41] Lu K. Otolaryngology Image Atlas. https://www.otoscape.com/image-atlas.html.

[42] Szegedy C., Vanhoucke V., Ioffe S., Shlens J., Wojna Z. (2016). Rethinking the Inception Architecture for Computer Vision. Proceedings of the 2016 IEEE Conference on Computer Vision and Pattern Recognition (CVPR).

[43] Detlefsen N., Borovec J., Schock J., Jha A., Koker T., Di Liello L., Stancl D., Quan C., Grechkin M., Falcon W. (2022). TorchMetrics—Measuring Reproducibility in PyTorch. J. Open Source Softw..

[44] Obukhov A., Seitzer M., Wu P.W., Zhydenko S., Kyl J., Yu-Jing Lin E. (2026). Torch-Fidelity: High-Fidelity Performance Metrics for Generative Models in PyTorch, Version v0.4.0.

[45] Gea S.L.R., Decraemer W.F., Funnell W.R.J., Dirckx J.J.J., Maier H. (2010). Tympanic Membrane Boundary Deformations Derived from Static Displacements Observed with Computerized Tomography in Human and Gerbil. J. Assoc. Res. Otolaryngol..

[46] Fisher E.W., Pfleiderer A.G. (1992). Assessment of the Otoscopic Skills of General Practitioners and Medical Students: Is There Room for Improvement?. Br. J. Gen. Pract..

[47] Wickens B., Lewis J., Morris D.P., Husein M., Ladak H.M., Agrawal S.K. (2015). Face and Content Validity of a Novel, Web-Based Otoscopy Simulator for Medical Education. J. Otolaryngol. Head Neck Surg..

[48] Davies J., Djelic L., Campisi P., Forte V., Chiodo A. (2014). Otoscopy Simulation Training in a Classroom Setting: A Novel Approach to Teaching Otoscopy to Medical Students. Laryngoscope.

[49] Lee D.J., Fu T.S., Carrillo B., Campisi P., Forte V., Chiodo A. (2015). Evaluation of an Otoscopy Simulator to Teach Otoscopy and Normative Anatomy to First Year Medical Students. Laryngoscope.

[50] Oyewumi M., Brandt M.G., Carrillo B., Atkinson A., Iglar K., Forte V., Campisi P. (2016). Objective Evaluation of Otoscopy Skills Among Family and Community Medicine, Pediatric, and Otolaryngology Residents. J. Surg. Educ..

[51] De Greef D., Goyens J., Pintelon I., Bogers J.-P., Van Rompaey V., Hamans E., Van de Heyning P., Dirckx J.J.J. (2016). On the Connection between the Tympanic Membrane and the Malleus. Hear. Res..

[52] Akenine-Möller T., Haines E., Hoffman N., Pesce A., Iwanicki M., Hillaire S. (2018). Real-Time Rendering.

